# Characteristics and Outcomes of Adolescents (15–18 Years) with Chronic Myelogenous Leukemia (CML) in Chronic Phase: The Experience of the International Registry of Childhood CML ^†^

**DOI:** 10.3390/cancers18121959

**Published:** 2026-06-16

**Authors:** Frédéric Millot, Morgane Froment, Lisa Durocher, Markus Metzler, Barbara De Moerloose, Silvia Regina Brandalise, Marina Borisevich, Petr Sedlacek, Adalet Meral Gunes, Birgitte Lausen, Antonio Molines Honrubia, Gordana Jakovljevic, Birgitta Versluys, Krzysztof Kalwak, Ana Hraskova, Meinolf Suttorp

**Affiliations:** 1 Inserm CIC 1402, University Hospital, 86000 Poitiers, France; lisa.durocher@chu-poitiers.fr; 2 Department of Pediatric Hematology-Oncology, University Hospital Poitiers, 86000 Poitiers, France; morgane.froment@gmail.com; 3 Pediatric Oncology and Hematology, Department of Pediatrics and Adolescent Medicine, 10 University Hospital Erlangen, 91054 Erlangen, Germany; 4 Department of Pediatric Hematology-Oncology and Stem Cell Transplantation, Ghent University Hospital, 9000 Ghent, Belgium; barbara.demoerloose@uzgent.be; 5 Boldrini’s Children Center, Campinas 13083-210, SP, Brazil; silvia@boldrini.org.br; 6 Belarusian Research Centre for Paediatric Oncology, Haematology and Immunology, 223053 Minsk, Belarus; mborisevich76@gmail.com; 7 Department of Pediatric Hematology and Oncology, University Hospital Motol, Charles University, 150 06 Prague, Czech Republic; petr.sedlacek@lfmotol.cuni.cz; 8 Pediatric Hematology and Oncology Hospital Görükle, Nilüfer 16120, Bursa, Turkey; 9 Rigshospitalet, National University Hospital, 2200 Copenhagen, Denmark; birgitte.lausen@regionh.dk; 10 Department of Pediatric Hematology, CHU Insular Materno-Infantil Las Palmas De Gran Canaria, 35016 Las Palmas de Gran Canaria, Spain; amolhon@gobiernodecanarias.org; 11 Department of Pediatric Hematology Oncology, Children’s Hospital, 10000 Zagreb, Croatia; gordanajakovljevic@yahoo.com; 12 Pediatric Oncology and Hematology, Wilhelmina Children’s Hospital/UMC Utrecht, 3584 EA Utrecht, The Netherlands; a.b.versluijs-2@prinsesmaximacentrum.nl; 13 Department of Pediatric Hematology, Oncology and Bone Marrow Transplantation, Wroclaw Medical University, 50-367 Wroclaw, Poland; krzysztof.kalwak@gmail.com; 14 Department of Pediatric Oncology, University Children’s Hospital, 833 40 Bratislava, Slovakia; hraskova.andrea@gmail.com; 15 Pediatric Hemato-Oncology, Medical Faculty, Technical University Dresden, 01069 Dresden, Germany; meinolf.suttorp@ukdd.de

**Keywords:** adolescents, chronic myeloid leukemia, tyrosine kinase inhibitor, imatinib

## Abstract

*Background:* Chronic myeloid leukemia (CML) is a rare disease in children and adolescents. Discrepancies have been reported regarding the outcomes of adolescents, most of whom have been studied with younger or older patients not separately. *Methods:* The International Registry of Pediatric CML (I-CML-Ped Study) retrospectively and prospectively enrolled 614 patients less than 18 years old. Data from adolescents (15–18 years) with CML in chronic phase (CML-CP) were analyzed and compared to data from children less than 15 years old enrolled during the same period. *Results:* In total, 132 out of 144 adolescents presented with CML-CP: palpable spleen was reported in 67%, and the median leukocyte count was 181 G/L [interquartile range, 70–327]. Responses to treatment and outcome were analyzed in 124 adolescents with imatinib as first-line treatment: the cumulative incidence of major molecular responses (MMR) at months 12 and 36 was 33.4% (95% confidence interval CI, 24.9–41.9) and 79.8% (95% CI, 71.8–87.7), respectively. With a median follow-up of 4 years (95% CI: 3.5–4.9), 49 (40%) of these 124 patients were switched to another treatment, 11 (9%) patients progressed to the advanced phase, and the five-year overall survival rate was 95.2% (95% CI, 90.8–99.9%). We did not find significant statistical differences between these adolescents and younger children receiving imatinib in terms of response (complete cytogenetic response, MMR) and outcome (progression-free survival, overall survival). *Conclusions*: Response to treatment and outcomes in adolescents with CML-CP in the tyrosine kinase inhibitor era are satisfying without statistically significant difference from results observed in younger children.

## Simple Summary

Chronic myeloid leukemia (CML) is a rare disease in adolescents. We compared characteristics, responses to treatment and outcomes of 132 adolescents (15–18 years of age at diagnosis) in the chronic phase enrolled in the International Registry of Pediatric CML to children less than 15 years enrolled during the same period of time. Significantly lower frequency of splenomegaly and lower median of leukocyte counts were observed in adolescents compared to younger children. We did not find significant statistical differences between 124 adolescents and younger children receiving first-line imatinib in terms of complete cytogenetic response, major molecular response, progression-free survival and overall survival. The 5-year overall survival rate of our cohort of adolescents receiving imatinib compares favorably with that reported in adults.

## 1. Introduction

Chronic myeloid leukemia (CML) is a myeloproliferative malignancy characterized by the presence of the Philadelphia chromosome (Ph), a reciprocal balanced translocation t(9;22)(q34;q11), resulting in the synthesis of the BCR::ABL1 oncoprotein with constitutive tyrosine kinase activity. CML is a very rare disease in pediatric cohorts and exhibits a more aggressive presentation than in adults because of a higher tumoral burden in this age range [1]. The characteristics and outcome of adolescents aged between 15 and 18 with CML have not previously been described separately in the tyrosine kinase inhibitors (TKI) era and few data regarding adolescents are available in part due to inclusion of adolescents with young adults (AYAs) from 15 to 29 years old and 16 to 29 years old in Pemmaraju et al. and Kalmanti et al. studies, respectively, or from 9 to 19 years of age in the study by Dou et al. [2,3,4]. Adolescents represent a real challenge for practitioners due to uncertainties in terms of clinical and biological characteristics, treatment choices, adherence to treatment, and outcomes. To the best of our knowledge, the International Registry of Chronic Myeloid Leukemia for Children and Adolescents (I-CML-Ped Study; www.clinicaltrials.gov NCT01281735) represents the largest cohort for patients under 18 years of age diagnosed with CML, providing us with an opportunity to focus on adolescents. In the present work, we focused on adolescents defined as those aged 15 to 18 years at the time of diagnosis of CML in chronic phase (CML-CP) because this age range represents a transition between childhood and adulthood care when teenagers increasingly take responsibility for the management of their disease. Adherence to treatment at different ages is strongly dependent on the parents’ consistent application of medication in very young patients and the patient’s desire for autonomy and adherence in adolescents [5]. The impact of these influencing factors is usually only anecdotally reported and hardly ever quantified [6]. For that reason, we selected adolescents aged 15 and 18 years old enrolled in the I-CML-Ped Study with the aim of comparing their characteristics, responses to treatment and outcomes to those of children younger than 15 years.

## 2. Patients and Methods

The international registry for childhood CML (I-CML-Ped Study registered at www.clinicaltrials.gov NCT01281735) was set up in order to better describe this disease and the outcome in children and adolescents under 18 years of age at diagnosis of BCR::ABL1-positive CML, whatever the stage (chronic phase, advanced phase) of the disease. Worldwide enrollment was done retrospectively from 2000 to 2010 and thereafter prospectively from 17 participating countries. The I-CML-Ped Study was approved by the French Data Protection Authority (CNIL: Comité National de l’Informatique et des Libertés, Paris, France) in December 2010 (number 9100031) and the Advisory Committee on Information Processing in Health Research (CCTIRS: Comité Consultatif pour le Traitement de I’Information en matière de Recherche dans le domaine de la Santé, Paris, France) in October 2009 (number 09.401 bis) and conducted in accordance with the Declaration of Helsinki. Written informed consent was obtained from the children and/or their legal guardians.

Gender, age, performance status, Sokal (for patients aged less than 45 years) and Eutos Long Term Survival (ELTS) risk scores, clinical features, blood counts, type of transcripts and cytogenetic assessments were collected at diagnosis [7,8,9,10]. Cytogenetic and molecular responses were assessed until achievement of CCR and MMR on a trimestrial basis. Cytogenetic and molecular responses to treatment, rate of progression to accelerated phase or blast crisis and overall survival (OS) were analyzed throughout the study. Response to treatment was defined according to the period of time to the effective European LeukemiaNet (ELN) criteria [11,12]. Complete cytogenetic response (CCyR) to treatment was defined as the absence of Philadelphia-chromosome-positive cells in at least 20 analyzed bone marrow metaphases. BCR::ABL1 transcript levels in the peripheral blood or in bone marrow were determined by local standardized laboratories using quantitative reverse transcriptase-polymerase chain reaction (RT-qPCR) and expressed according to the International Scale (IS) [13]. Major molecular response (MMR) and deep molecular response (DMR) were defined as a ratio of BCR::ABL1/ABL1 less than 0.1% and less than 0.01% (MR^4^), respectively [10]. If treatment-free remission was attempted, molecular relapse was defined as a loss of MMR at any time. Transcript types and BCR::ABL1 kinase domain mutations analysis were determined as previously reported [9,14].

Time to achieve CCyR or MMR was calculated from the treatment’s start to the date of CCyR or MMR achievement. Progression-free survival (PFS) was measured from the start of the treatment to the date known to be the date of progression, death, or the latest follow-up date, whichever occurred first. OS was measured from the date treatment was started to the date of death, whatever the cause, or to the date of last follow-up.

Adverse events (AEs) related to imatinib treatment were reported and censored for those that appeared before the start of imatinib or after the end of imatinib treatment. AEs were graded according to the Common Terminology Criteria for Adverse Events (CTCAE) scale version 4 until 2017, then after according to the fifth version [15,16].

Differences between variables were assessed by the Chi-2 test or Fisher’s exact test for categorical variables and the Student’s *t*-test or the Mann–Whitney U test for continuous variables, as appropriate.

Survival probabilities were estimated using the Kaplan–Meier method and compared with the log-rank test. Cumulative incidence functions accounting for death as a competing event were estimated using the Aalen–Johansen method and compared between groups using Gray’s test. Follow-up of patients was not censored at the time of switching to other treatments, including hematopoietic stem cell transplantation (HSCT). Clinical and biological characteristics at diagnosis were analyzed as predictors of major molecular response (MMR) using a Cox proportional hazards model.

All statistical tests were two-sided, and a *p*-value ≤ 0.05 was considered statistically significant. No imputation was performed for missing data. Analyses were performed using R software, version 4.0.4 (R Foundation for Statistical Computing, Vienna, Austria).

## 3. Results

Among the 614 patients with CML under 18 years old at diagnosis recruited in the I-CML-Ped Study from January 2011 to March 2021, we identified 144 (23.4%) adolescents (15–18 years of age at diagnosis of CML) with sufficient relevant data. Among them, according to the ELN criteria, 132 (92%), seven (5%), and five (3%) patients presented in chronic phase, accelerated phase or blast phase, respectively.

Clinical and biological characteristics of the 132 adolescents diagnosed with CML-CP are detailed in Table 1 and compared to those of the 403 children less than 15 years old diagnosed with CML. As is the case in younger children, adolescents with CML are predominantly male. Most adolescents were fully active at diagnosis with a performance status score comparable to that of younger children (Table 1). Ninety-two percent were symptomatic at diagnosis. The most frequent symptoms were pain (36%), asthenia (33%), and weight loss (22%) (Supplemental Figure S1). CML was diagnosed fortuitously in 14% of the adolescents. Significantly lower frequency of palpable spleen and a lower rate of leukocyte counts were observed in adolescents, whilst a significantly higher hemoglobin level was observed compared to younger patients (Table 1). Distribution across the three risk categories according to the ELTS scoring system was similar in adolescents and younger children, with the majority (68%) of the adolescents allocated to the low-risk group. By contrast, the majority (69%) of the adolescents were categorized as intermediate and high risk according to the Sokal scoring system (Table 1). Philadelphia chromosome variants and additional chromosomal abnormalities (ACA) were rarely found (2% and 3%, respectively) in the 128 adolescents with available karyotype at diagnosis (Table 1). Similarly to younger children, the typical BCR::ABL1 transcript e14a2 (b3a2) was predominant (55%) in the 98 assessed adolescents (Table 1).

**Figure 1:**
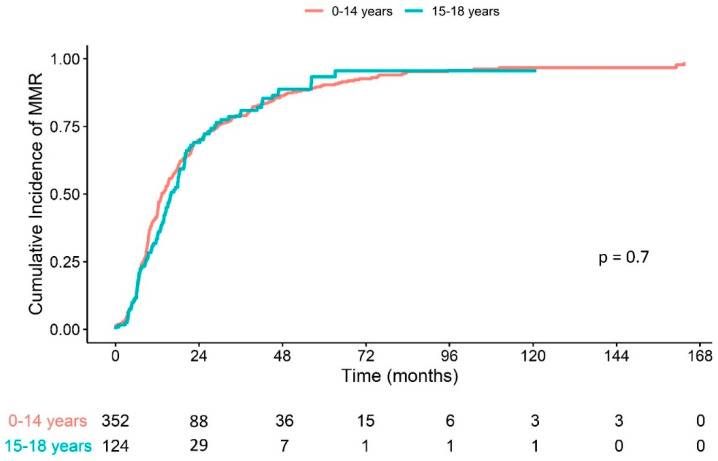
Cumulative incidence of major molecular response (MMR) in adolescents and children less than 15 years old treated with front-line imatinib.

Among the 132 adolescents with CML-CP at diagnosis, 124 were treated with imatinib as first-line treatment; the remaining ones were initially treated with dasatinib (n = 1), nilotinib (n = 3), bosutinib (n = 1) or interferon-alpha (n = 1) or a combination of interferon-alpha and cytosine arabinoside (n = 2). Treatment details, including the different switches among the 132 adolescents, are summarized in Supplementary Figure S2. The 124 (94%) adolescents receiving first-line imatinib therapy were considered for analysis of the response to treatment and outcome, and were compared to the 358 (89%) children less than 15 years old, likewise treated with imatinib as first-line treatment. The initial daily total dose of imatinib ranged from 200 to 800 mg, corresponding to a median initial daily dose of 247 mg/m^2^ (interquartile range IQR, 221–289 mg/m^2^), whilst patients less than 15 years old received a significantly (*p* < 0.0001) higher dose (median 288 mg/m^2^, IQR: 259–328). The BCR::ABL1/ABL1 ratio at 3 months of imatinib treatment was <10% in 54% of 91 adolescents with available data and 61% of 245 patients less than 15 years of age with available data (*p* = 0.2213). MMR achievement at 12 months and 36 months is reported in Table 2. Among the 124 adolescents receiving first-line imatinib therapy, 99 (79.8%) achieved MMR at the end of follow-up (under imatinib n = 77 or after a switch n = 22) within a median time of 16 months (95% CI, 14–19.6 months). The cumulative incidence of MMR in adolescents was similar to that of younger patients (Figure 1). In 108 out of the 124 adolescents receiving front-line imatinib, the ELTS score was available for multivariable analysis. The only factors associated with failure to achieve MMR were the intermediate risk stratification according to the ELTS score and leukocyte count greater than 100 G/L (Supplementary Table S1).

Among the 124 adolescents receiving first-line imatinib therapy, 13 patients attempted to discontinue imatinib: at last follow-up, four adolescents treated with imatinib for 2.8 to 9.2 years with sustained MR^4^ for 1 to 7 years were free of treatment 8.4 months to 11 years after discontinuation. The nine remaining adolescents treated with imatinib for a median duration of 40.16 months (range, 12–108 months) but without sustained MR^4^ lost their response and resumed TKI after a median duration of discontinuation of 4 months (range, 2 to 9 months).

Among the 124 adolescents treated with first-line imatinib, at least one AE during this treatment was reported in 57 (46%) of them. In most cases, toxicity was mild to moderate, while grade 3 and 4 AEs were recorded in 27 (22%) and four (3%) patients, respectively. Neutropenia was the most commonly observed toxicity (7%). Toxicity led to transient treatment discontinuation and/or dose adjustment in 17 (14%) patients with blood count and/or metabolic abnormalities or musculoskeletal symptoms.

**Figure 2:**
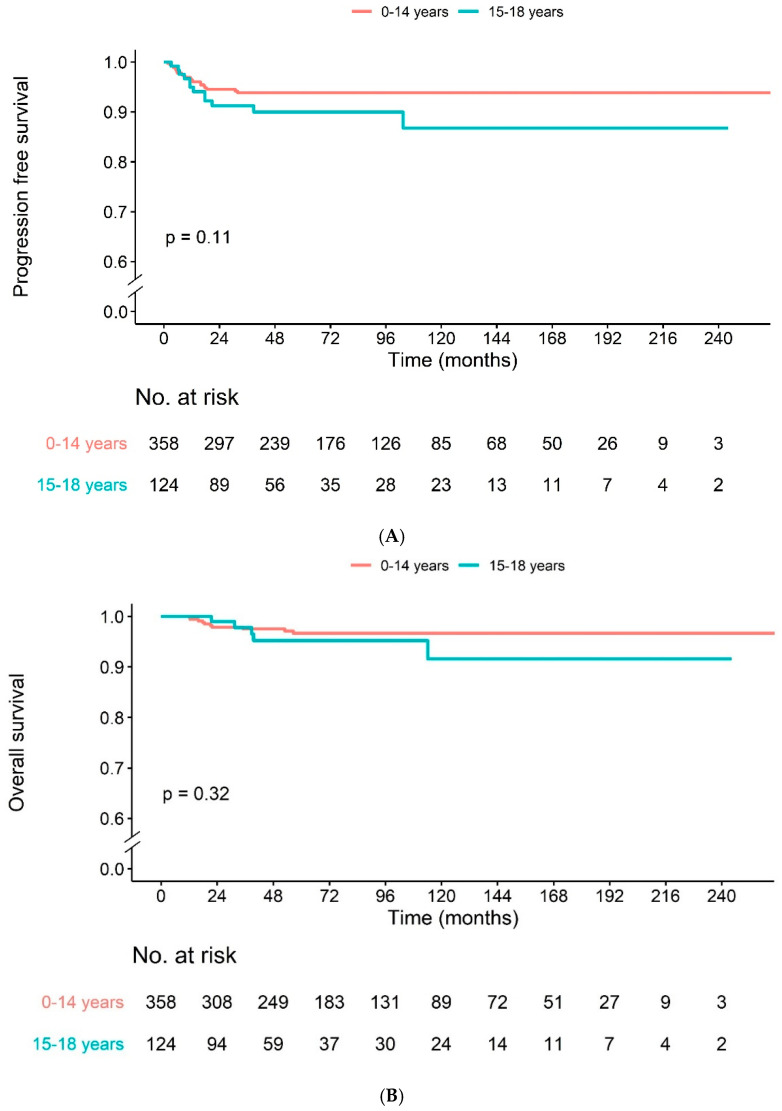
Outcomes of adolescents and children less than 15 years old treated with first-line imatinib. (**A**) Progression-free survival; (**B**) overall survival.

In patients receiving imatinib as first-line treatment, progression to an advanced stage of the disease was observed in 11 (9%) of the 124 adolescents and in 18 (5%) younger children after a median follow-up period of 11 months (range: 3 to 103) and 8.5 months (range: 2 to 32), respectively. However, no statistically significant difference in progression-free survival (PFS) was found between the adolescent and younger patient cohorts (Figure 2).

Among the 124 adolescents, five deaths were recorded (treatment related mortality post transplantation n = 4; progression of the disease n = 1). Overall survival (OS) did not significantly differ between adolescents and younger patients (Table 2, Figure 2).

## 4. Discussion

It has been established that children and adolescents tend to present with more aggressive features at diagnosis of CML [1,4,17,18]. Scant data are available in adolescents, and inconsistencies in terms of cytogenetic and molecular response have been reported in this population. However, these studies reported on adolescents from cohorts with a wide age range, including patients aged from 15 or 16 to 29 years or from the age of theoretical onset of puberty (9 years for girls, 11 years for boys) to 19 years [2,3,4]. The International Registry of Pediatric CML (I-CML-Ped Study) is based on an international collaboration with the aim of better describing this very rare disease in cohorts of patients less than 18 years of age [19]. The I-CML-Ped Study enabled retrospective analysis of a large cohort of adolescents between 15 and 18 years of age at diagnosis of CML-CP in the tyrosine kinase inhibitor (TKI) era.

As previously observed in pediatric, AYAs and older patients’ series, male preponderance is reported in the population of adolescents enrolled in the I-CML-Ped Study [1,2,3,4,20,21]. Pain, asthenia and splenomegaly were the predominant symptoms and clinical signs in the 132 adolescents of the I-CML-Ped Study diagnosed with CML-CP. These adolescents presented with a significantly lower frequency (67%) of splenomegaly compared with younger children (79%) enrolled in the I-CML-Ped Study, whilst this frequency appeared higher than in the older age group (53.5%), a finding suggesting decreased frequency of palpable spleen according to the ages of patients diagnosed with CML-CP [21]. The same observation applies to the white blood cell counts at diagnosis, with a higher amount in younger children and a progressive decrease with age [2,20,21]. These findings reflect a higher tumor burden in younger patients at diagnosis of CML in CP. We observed a significantly lower hemoglobin level (mean: 9 g/dL +/− 2 SD) at diagnosis among children less than 15 years old compared to adolescents (mean: 11 g/dL +/− 2 SD), whilst this level seemed to approach the median hemoglobin level (11.7 to 12.5 g/dL) reported in adults [21]. As in children less than 15 years of age, the clinical and biological characteristics at diagnosis of adolescents between 15 and 18 years of age seem to be more aggressive than those reported in older patients [20,21].

In the present work, 39% of adolescents and 45% of children less than 15 years of age were allocated to the high-risk group according to the Sokal risk score. This proportion is markedly higher than those reported in AYAs (20 to 31%) and adults (24.7%) [3,20]. By contrast, taking into consideration the ELTS risk score, the proportion of our adolescents (9%) allocated to the high-risk group was quite similar to that (15%) reported in adults [22]. Current recommendations for patients less than 18 years old with CML-CP propose a second-generation TKI as front-line therapy in patients allocated to the ELTS high-risk group because of a lower PFS rate in these patients receiving imatinib [23].

A substantial rate of switches (40%) was observed in our 124 adolescents treated with first-line imatinib. Cumulative cytogenetic and molecular responses are in accordance with the results reported in previous prospective pediatric and adult trials reflecting the positive impact of such switches, including HSCT, on the long-term outcome of our adolescents [24,25,26,27]. HSCT remains a treatment option in some adolescents who do not respond well to several lines of TKI. Interestingly, the rates of cytogenetic and molecular responses to imatinib at different time points observed among our adolescents were not statistically different from those observed in children less than 15 years of age.

Among our patients treated with imatinib, while 9% of the adolescents and 5% of the children less than 15 years old progressed to advanced disease, this did not translate into significantly different PFS. Progression rates reported in adults and AYAs ranged from 6 to 7.4% and 6.5 to 14.2%, respectively [2,3,20,26]. As previously reported, while we observed lower prescribed median TKI doses in adolescents than in younger children, this did not translate into a difference in terms of response to the treatment [28]. It has been suggested that the underlying reason for the lower doses prescribed in adolescents can be explained by rounding to the lower dose in order to avoid the use of splitting tablets [28]. In line with this finding, when 57 patients with CML in chronic phase (median age 11.4 years, range 2.8–17.9) from 13 pediatric and adult Italian centers were retrospectively analyzed, the patients managed in adult centers received a lower median imatinib dose [29].

The 5-year OS rate (95.2%) in our cohort of adolescents receiving imatinib is similar to that of younger patients (96.7%) and compares favorably with those reported in comparative studies of AYAs (93 to 96.7%) and adults (93%), a finding suggesting that despite more aggressive features at diagnosis, adolescents tend to have outcomes comparable to those of older patients [2,3,20].

In this study, 12 adolescents performed a stopping attempt, which was successful in four who remained free of treatment with significant follow-up after imatinib discontinuation, while the nine remaining adolescents failed to achieve treatment-free remission. However, in these nine remaining adolescents, the criteria for discontinuation (sustained MR^4^ for at least 2 years and TKI for at least 3 years) had not been met [30,31].

## 5. Conclusions

Despite concerns that the outcome may be inferior in adolescents with CML-CP, both responses to treatment and outcome in adolescents with CML-CP appear favorable in the tyrosine kinase inhibitors era, and no significant difference was observed compared with younger children. Further studies comparing the characteristics and outcomes of adolescents to those of young adults are needed.

## Figures and Tables

**Table 1:** Baseline characteristics of 132 adolescents and 403 children less than 15 years old at diagnosis of chronic myeloid leukemia in chronic phase. The Karnofsky Scale is designed for patients aged 16 years and older, and the Lansky Scale for patients aged one year to less than 16 years of age.

	All Patients (n = 535)	0–14 Years (n = 403)	15–18 Years (n = 132)	*p* *
**Gender**, n (%)				0.0615
Boys	303 (57%)	219 (54%)	84 (64%)	
Girls	232 (43%)	184 (46%)	48 (36%)	
**Lansky/Karnofsky score**, n (%)				0.1978
<90	103 (21%)	84 (22%)	19 (17%)	
≥90	384 (79%)	290 (78%)	94 (83%)	
**Palpable spleen**, n (%)				0.0047
Yes	404 (76%)	316 (79%)	88 (67%)	
No	129 (24%)	85 (21%)	44 (33%)	
Median length below the costal margin (cm)	9 (4–15)	8 (4–14)	10 (5–18)	0.0040
**Sokal score** (<45 years), n (%)				0.0028
High	193 (43%)	150 (45%)	43 (39%)	
Intermediate	165 (37%)	132 (39%)	33 (30%)	
Low	88 (20%)	54 (16%)	34 (31%)	
**ELTS score, n (%)**				0.4645
High	59 (13%)	48 (14%)	11 (9%)	
Intermediate	96 (21%)	70 (20%)	26 (22%)	
Low	310 (67%)	231 (66%)	79 (68%)	
**WBC (10^9^/L) median (IQR)**	222 (95–353)	238 (102–360)	181 (70–327)	0.0177
**Hemoglobin level (g/dL) mean standard deviation**	10 ± 2	9 ± 2	11 ± 2	<0.0001
**Platelet count (10^9^/L) median (IQR)**	494 (337–778)	489 (347–799)	516 (287–721)	0.4128
**Karyotype**	n = 511	n = 383	n = 128	0.4620
Classical Ph+ translocation	483 (95%)	362 (95%)	121 (95%)	
Variant Ph+ translocation	7 (1%)	4 (1%)	3 (2%)	
ACA	21 (4%)	17 (4%)	4 (3%)	
**Transcript type**	n = 381	n = 283	n = 98	0.9345
e14a2 (b3a2)	199 (52%)	145 (51%)	54 (55%)	
e13a2 (b2a2)	123 (32%)	92 (33%)	31 (32%)	
Combination e14a2-e13a2	50 (13%)	39 (14%)	11 (11%)	
Atypical transcript e13a3 (b2a3)	9 (2%)	7 (2%)	2 (2%)	

* Comparison between adolescents aged 15–18 years and children under 15 years of age. Abbreviations: ACA: additional chromosomal abnormalities in Ph+ metaphases; IQR: interquartile range; WBC: white blood cell count.

**Table 2:** Outcome in patients treated with first-line imatinib (95% confidence interval).

	All (n = 482)	0–14 Years (n = 358)	15–18 Years (n = 124)
Overall survival rate at 5 years (%)	96.4 [94.5–98.3]	96.7 [94.6–98.7]	95.2 [90.7–99.9]
Progression-free survival at 12 months (%)	96.0 [94.2–97.8]	96.3 [94.4–98.3]	95 [91–99.0]
Major molecular response (MMR)	n = 476	n = 352	n = 124
Cumulative incidence at 12 months (%)	41.7 [37.2–46.2]	43.7 [38.5–49.0]	35.9 [27.2–44.5]
Cumulative incidence at 36 months (%)	79.3 [75.5–83.2]	79.0 [74.5–83.5]	80.9 [73.0–88.7]
Median time to achieve MMR (months)	13.8 [12.7–15.4]	13.0 [12.1–15.1]	15.7 [13.3–19.6]
Complete cytogenetic response (CCyR)	n = 478	n = 356	n = 122
Cumulative incidence at 12 months (%)	71.6 [67.5–75.7]	73.3 [68.6–78.0]	66.5 [58.0–75.1]
Median time to achieve CCyR (months)	6.8 [6.4–8.1]	6.8 [6.4–8.1]	6.9 [6.2–9.0]

## Data Availability

The data that support the findings of this study are available from the corresponding author on reasonable request.

## Supplementary Materials

The following supporting information can be downloaded at: https://www.mdpi.com/article/10.3390/cancers18121959/s1, Figure S1: Symptoms at diagnosis; Figure S2: Sankey diagram of treatment patterns; Table S1: Forest plot of factors influencing the achievement of major molecular response.
