# Supplementary material for: Characteristics and Outcomes of Adolescents (15–18 Years) with Chronic Myelogenous Leukemia (CML) in Chronic Phase: The Experience of the International Registry of Childhood CML
Source: Cancers (Basel). 2026 Jun 16;18(12):1959. doi: 10.3390/cancers18121959 (PMC13296705; doi:10.3390/cancers18121959)

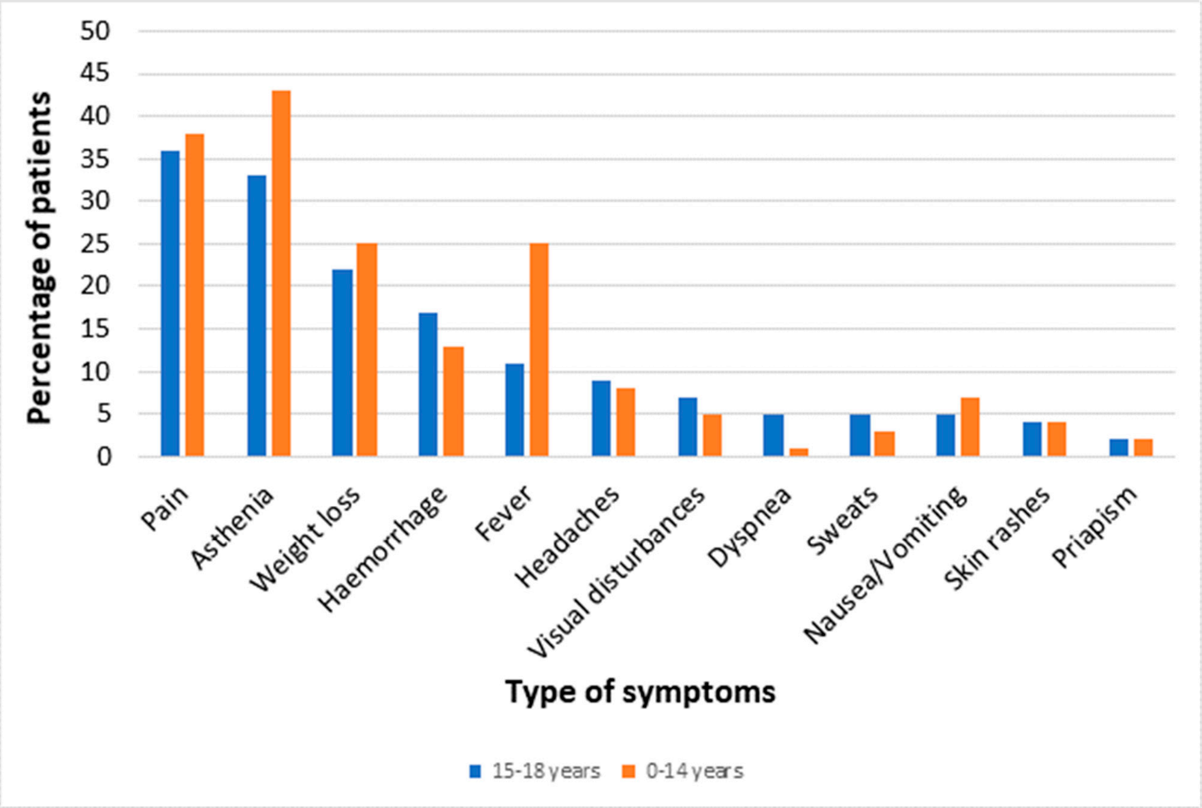

Supplementary Figure S1. Symptoms at diagnosis recorded in 122 (92%) of the 132 adolescents and 387 (96%) of the 403 children less than 15 years old with chronic myeloid leukemia in chronic phase.

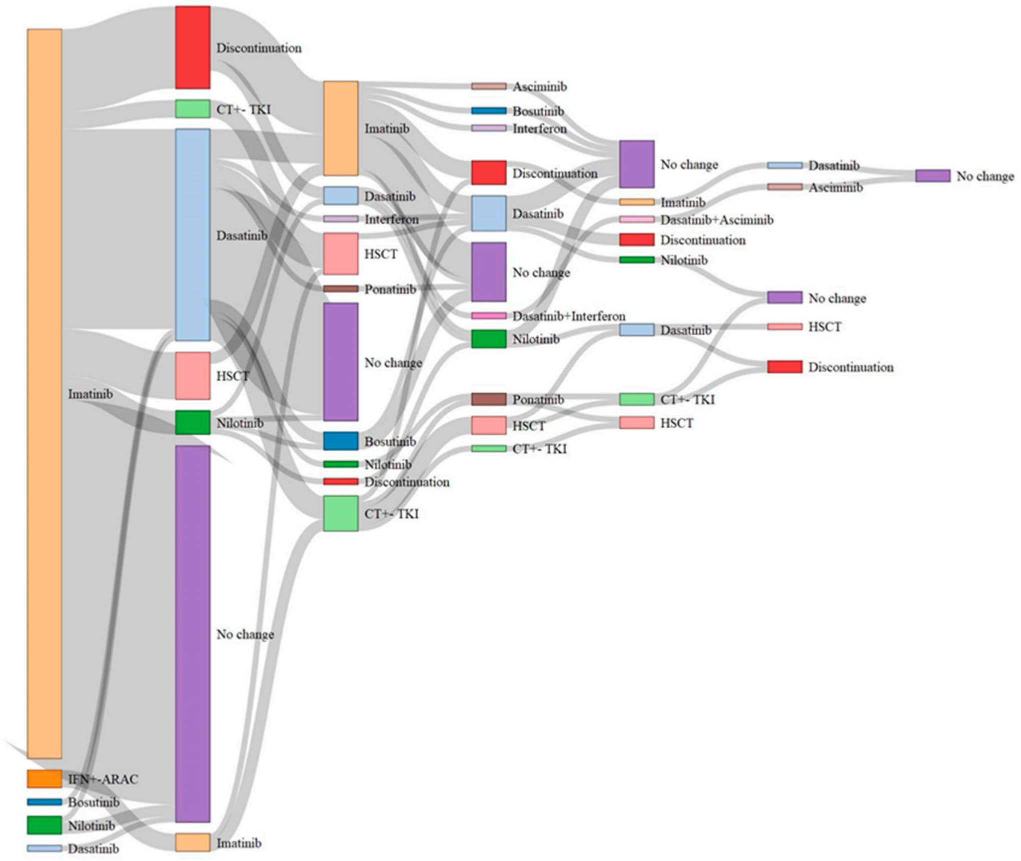

Supplementary Figure S2. Sankey diagram of treatment patterns (1st line to 6th line) of the 132 adolescents. Among the 124 adolescents treated with front-line imatinib, 49 (40%) patients were switched to another second-line treatment and received: dasatinib (n = 34: toxicity reasons n = 11, failure to achieve CHR, CCyR, MMR n = 12, progression n = 2, loss of response n=6, unknown n = 3), nilotinib (n = 4: toxicity reasons n = 1, failure to achieve CCyR or MMR n = 2, clinician choice n = 1), chemotherapy <u>+</u> ITK (n = 3; progression n=3), HSCT (n = 8, failure to achieve MMR or clinician choice n= 6, progression n = 2).

Supplementary Table S1. Forest plot: Factors influencing the achievement of major molecular response in 124 adolescents treated with front-line imatinib.

Variable		N	Hazard ratio		p
**ELTS**	Low	75		Reference	
	Intermediate	23		0.45 (0.24, 0.86)	0.01
	High	10		0.94 (0.43, 2.06)	0.88
**Leukocyte** (>100 versus ≤100)		108		0.56 (0.34, 0.94)	0.03
**Gender** (girl versus boy)		108		0.95 (0.58, 1.55)	0.84

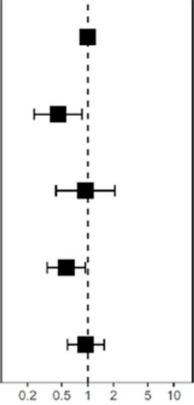

Supplement: Supplementary file 1 [file cancers-18-01959-s001.zip › cancers-4277101-supplementary.pdf]
